# Potential and Prospects of Continuous Polyhydroxyalkanoate (PHA) Production

**DOI:** 10.3390/bioengineering2020094

**Published:** 2015-05-29

**Authors:** Martin Koller, Gerhart Braunegg

**Affiliations:** 1 Office of Research Management and Service, c/o Institute of Chemistry, Research Group Interfaces, Division of Physical and Theoretical Chemistry, University of Graz, NAWI Graz, Heinrichstrasse 28/III, Graz A-8010, Austria; 2 ARENA-Association for Resource Efficient and Sustainable Technologies, Inffeldgasse 21b, Graz 8010, Austria; E-Mail: g.braunegg@tugraz.at

**Keywords:** bioreactor cascade, chemostat, continuous process, copolyester, extremophiles, inexpensive carbon sources, polyhydroxyalkanoates (PHA), process design, unsterile process

## Abstract

Together with other so-called “bio-plastics”, Polyhydroxyalkanoates (PHAs) are expected to soon replace established polymers on the plastic market. As a prerequisite, optimized process design is needed to make PHAs attractive in terms of costs and quality. Nowadays, large-scale PHA production relies on discontinuous fed-batch cultivation in huge bioreactors. Such processes presuppose numerous shortcomings such as nonproductive time for reactor revamping, irregular product quality, limited possibility for supply of certain carbon substrates, and, most of all, insufficient productivity. Therefore, single- and multistage continuous PHA biosynthesis is increasingly investigated for production of different types of microbial PHAs; this goes for rather crystalline, thermoplastic PHA homopolyesters as well as for highly flexible PHA copolyesters, and even blocky-structured PHAs consisting of alternating soft and hard segments. Apart from enhanced productivity and constant product quality, chemostat processes can be used to elucidate kinetics of cell growth and PHA formation under constant process conditions. Furthermore, continuous enrichment processes constitute a tool to isolate novel powerful PHA-producing microbial strains adapted to special environmental conditions. The article discusses challenges, potential and case studies for continuous PHA production, and shows up new strategies to further enhance such processes economically by developing unsterile open continuous processes combined with the application of inexpensive carbon feedstocks.

## 1. Introduction

Although continuous culture fermentation strategy has unquestioned advantages over established dis- and semi-continuous approaches, it is still anticipating its broad implementation at the industrial scale [1]. On a laboratory- to pilot-scale, this technique has already been successfully tested with intact active microorganisms, either free or immobilized. *Inter alia*, the production of the *Clostridia* products acetone, butanol and 2-propanol [2,3,4,5], ethanol produced by yeasts or *Klyveromyces marxianus* [6,7,8,9], lactic acid from various lactic acid bacteria [10,11,12,13], fungal citric acid [14,15,16], thermogelable polysaccharides (“curdlan”; [17]), different bacteriocins [18,19,20], enzymes [21,22], or biohydrogen [23] is described in the literature.

It has to be emphasized that fundamental differences exist between the possibilities to run continuous processes for formation of, on the one hand, extracellular products as listed in the prior paragraph, and, on the other hand, as the topic of this review, for intracellular products like microbial polyhydroxyalkanoates (PHA). In the case of extracellular products, continuous processes can be conveniently designed by using immobilized cells acting as fixed whole-cell biocatalysts. The substrate in a defined feed stream is continuously metabolized by the biocatalytically active layer, and a cell-free product stream is continuously released from the bioreactor set-up. Such approaches are well-known from beverage industry; e.g., continuous beer, wine and cider production is reported on a semi-industrial scale by carrying out the fermentation by immobilized yeast cells, either fixed on the bioreactor’s inner surface, or agglomerated with each other. Another example for successful continuous cultivation to gain extracellular products is the conversion of starch to lactic acid by *Lactobacillus amylovorus* in a membrane recycle reactor set-up [13]. These examples are in clear contrast to the PHA-case; here, a sufficient number of active cells have to be formed under nutritionally balanced conditions in a first stage; in a second stage, these cells have to accumulate the biopolymer as intracellular product. PHA serve as intracellular carbon and energy storage, and provide the microbial cells with an advantage for survival under conditions of stress and/or starvation. Dependent on the enzymatic machinery of the cells, either short-chain-length PHA (*scl*-PHA) or medium-chain-length PHA (*mcl*-PHA) are accumulated, classically provoked by limitation of a growth-essential compound plus a surplus of exogenous carbon source. [24] Although even efforts for solid-state PHA production are reported, these processes resulted in modest productivity [25]; the two-step process characteristic for PHA production can only be efficiently performed in submerse cultures; hence, by cultivating non-immobilized, free active microbial biomass. Also, the morphology of the applied production strain determines its applicability for different modes for continuous production; fungal mycel-forming strains are often difficult to be applied as free cells in continuous mode due to the risk of blocking of the tubular bioreactor system, a risk that is considerably lower in the case of PHA accumulation organisms, typically not exceeding dimensions of a few micrometers in length. Mycel-forming organisms should rather be immobilized if operated continuously [14,15,16].

As an extension of a previous review article on continuous chemostat PHA production [26], the article at hand also shines a light on engineering aspects (fermentation, downstream processing), discussed continuous techniques for enrichment of PHA-accumulating microbial consortia, and gives insights on new cost-efficient approaches by applying extremophile production strains, which can be long-term cultivated at minor sterility requirements. Some crucial aspects that are cumbersome for a broad industrial implementation of continuous fermentation should be accentuated; together with suggestions to minimize or even overcome the problems, these aspects are listed in Table 1.

**Table 1:** Challenges in continuous fermentation and means to overcome them (adapted and extended from [26]).

Risk/Challenge	Remedy
Typically mesophile organisms are implemented in large-scale biotechnological production processes. Continuous cultivation of such organisms implies a substantial risk of microbial contamination; this can endanger whole fermentation batches, and, consequently, causes extensive economic loss.	Careful handling! High operational skills of staff! Application of extremophile production strains (e.g., thermophiles, osmophiles), which are robust, and outperform the growth rate of microbial competitors! Continuous antibiotic feed (generally not recommended!)
Long-term stability of production strain not assured	Strain improvement by genetic engineering. Application of robust production strains, which are rigid enough to withstand the shear forces generated by peristaltic pumping. Application of microbial fermentation broth that can conveniently be pumped (impedes the application of mycelia-forming species!)
Coming from the bioreactor’s interior, microbes may reach the tank of sterile medium; by subsequent conversion of substrate compounds, this can change the feed composition	Taking appropriate technical precautions, such as interrupting the liquid path in tubes by air barriers in which the medium drops, or by implementing thermo-traps (heating of the medium), *etc.* *Note*: Medium quality needs to be verified after the thermo-trap: precipitation or destruction of heat labile components has to be avoided.
Dropping of the media into the bioreactor’s interior (cultivation broth) results in small nutrient pulses and thus locally inconsistent nutrient concentrations. The same goes for locally fluctuating pH-values by acid or hydroxide pulses to maintain the pH-value. This prevents real “steady state” conditions.	Reduction of the volume ratio droplets/fermentation broth (small droplets favorable)
Highly fragile microbial cells can get disrupted by agitation and aeration	If possible, no excessive agitation and aeration. Application of robust microbial strains. Application of special stirrer (impeller) systems, which generate less shear stress.
Cell growth on the inner walls or other surfaces (e.g., baffles, probes, *etc.*) of the bioreactor during long-term operation	Equipping the reactor wall with a hydrophobic surface by application of e.g., silanes. “Close clearance” of inner walls by stirrers operating close to the wall
If mixing does not occur completely uniform, true “steady state” conditions are not warranted	Application of advanced adapted mixing systems (encompasses stirrer, sparger, baffles)
Instable reactor volumes by foaming, resulting in an overflow of fermentation broth	Application of effective antifoam agents tailored for the substrate-strain combination. Mechanical foam suppression by “anti-foam centrifuge”. Integrated solution: Application of a mass controlled pump (“mass-stat regime”) in combination with foam sensors and antifoam solutions.
Process separated in different phases (formation of secondary bio-product PHA after autocatalytic phase of biomass formation): optimum composition of the feed stream varies for the two different phases	Switch from single- to multistage continuous processes

## 2. Continuous *vs.* Discontinuous PHA Production

For efficient large-scale production, PHA-producing organisms have to be cultivated under controlled conditions in closed bioreactors, where stability of process parameters (pH-value, temperature, oxygen supply, substrate concentration) can be warranted, and monoseptic conditions are guaranteed by excluding microbial competitors [27]. As exhaustively described in literature, PHA production encompasses two easily distinguishable phases for most production strains: first, a sufficient concentration of catalytically active biomass with only minor quantity of accumulated PHA is produced in a nutritionally balanced growth medium. In a second phase, nutritional stress is provoked by restricting the supply of a growth-essential nutrient (e.g., nitrogen, phosphate, oxygen, *etc.*) resulting in the deviation of the carbon flux from biomass production towards PHA accumulation. In this second phase, biomass formation is negligible if compared to PHA accumulation [28,29,30].

Different operation modes are described for PHA production. Among them, discontinuous fed-batch strategies are most widely used at pilot- and industrial-scale [31]. Here, all substrates are supplied to the system according to their consumption by the active biomass. Cell harvest occurs in batch mode only at the end of the cultivation batch, normally after *in situ* biomass pasteurizing in the bioreactor. Such fed-batch processes are generally stable and, after elaborating reliable and detailed fermentation protocols for the production processes, they are well reproducible. As the downside, discontinuous processes feature limited productivity (amount of PHA produced per reactor volume and time—the cost-decisive factor for industrial PHA production); this is mainly caused by the “dead time” needed for preparation and post-treatment (“revamping”) of the bioreactor. In addition, product quality can fluctuate between different batches; this fluctuating product quality, e.g., in terms of molar mass distribution, is even more striking for biopolymer production than for well-established “simple” chemical polymerization processes to produce petrol-based plastics. A molar mass distribution as narrow as possible is desired to provide a product as uniform and homogenous as possible; further, high molar masses are favorable due to the fact that during PHA processing, e.g., by melt extrusion or injection molding, significant losses of molar mass are commonly observed, thus negatively impacting the material quality [32].

Furthermore, in the case of such substrates that reveal growth-inhibiting effects on the cells already at low concentration levels, it is challenging to obtain high productivity in discontinuous systems. Such substrates, typically fatty acids, are especially needed for *mcl*-PHA production by bacteria from the pseudomonad group [33,34,35,36,37,38]. As detailed later, continuous cultivation strategies provide a solution for this problem.

It is known that continuous cultivation regimes in chemostats (short for “chemical environment is static”) can guarantee growth of microorganisms under defined nutrient limitations for extended time periods, and, a long-term genetic stability of the organism provided, can result in both high productivities and constant product quality. As soon as steady-state conditions are reached, the concentration of biomass, PHA and all substrates is kept constant under such chemostat regimes [38,39,40]. Harvest of biomass that harbors a desired PHA-mass fraction also occurs continuously in chemostat regimes, which constitutes another considerable advantage to batch-processes. This can be visualized by the manageable quantities of PHA-rich biomass that permanently accrue for subsequent product recovery; these quantities can conveniently be processed in rather small facilities. This is in clear contrast to the enormous lot of PHA-rich biomass that accrues for one moment to the other after harvest of a batch, which requires large facilities for product recovery.

As pivotal process-engineering parameters of continuous processes, dilution rate *D* and residence time τ need to be explained. *D* (1/h) describes the quotient of the flow rate *F* (L/h) and the bioreactor’s volume *V* (L), whereas τ (h) is calculated as the inverse number of *D*, or as the quotient of *V* and *F*, respectively [38,39,40].

Apart from enhanced productivity and product quality (composition, molar mass and PDI of PHA), chemostat processes are also suitable to elucidate the physiological background of bioprocesses; kinetics of cell growth and PHA formation under constant environmental conditions, and the impact of changing conditions on the kinetics and on product properties, can conveniently be investigated [39]. This way, the optimization of nutritional media composition can easily be accomplished in chemostat processes by changing concentrations of single nutrients in the feed stream during steady-state, and monitoring the resulting reaction of microbial kinetics (growth rate, product formation rate, yields) [41].

Table 2 summarizes the discussed benefits arising from continuous PHA production.

**Table 2:** Advantages of continuous PHA-production.

Criterion	Benefit	References
Investment costs for bioreactor	Due to higher volumetric productivity in continuous processes, (fed)batch cultivation requires large bioreactor facilities to generate the same output per time; continuous production contributes to lower investment costs by resorting to smaller operation facilities	[32]
Time demand	No “dead time” needed for pre- and post-treatment (“re-vamping”) of bioreactor	[32]
Investment costs for downstream processing	Manageable quantities of PHA-rich biomass accrue continuously. Downstream processing (e.g., extraction) of crude product stream can be accomplished continuously in smaller (cheaper!) recovery facilities.	[32,42]
Labor intensity	Higher for (fed)batch processes; not too much effort needed from staff during continuous operation as soon as steady-state conditions are reached.	[32]
Product quality	Higher consistency and uniformity of product quality (molar mass distribution, distribution of monomeric building blocks, thermal properties)	[43,44,45,46]
Triggering of polyester composition	Easier in continuous processes by possibility to exactly trigger the ratio between main- and co-substrates in the continuous feed stream. In multi-stage continuous processes: Possibility to design blocky structured polyesters consisting of soft- and hard segments.	[43,44,46,47,48]
Triggering molar masses	The applied dilution rate *D* significantly impacts the molar mass of PHA (molar mass direct proportional to *D*)	[49]
Making toxic substrates better accessible to the production strain	Toxic substrates can be continuously supplied exactly in accordance to their conversion by the cells. Thus, inhibiting concentration are never reached, actual zero concentration in cultivation medium. In addition, one can even profit from dual nutrient limited (DNL) growth conditions. *Note*: The acceptable substrate concentration depends on the strain properties (substrate affinity, Ks) and the specific growth rate µ (or *D* at steady-state, respectively) at which the strain is cultured. Excessively increasing *D* can cause a wash-out of the culture because of the increased substrate toxicity which by itself reduces the specific growth rate!	[33,43,44,50,51]
Convenient method for medium development and optimization	Fast reaction of steady-state culture kinetics to changing process parameters such as substrate concentrations triggered by pulse, shift or transient changes, temperature, pH-value, *etc.*	[41,52]

**Figure 1:**
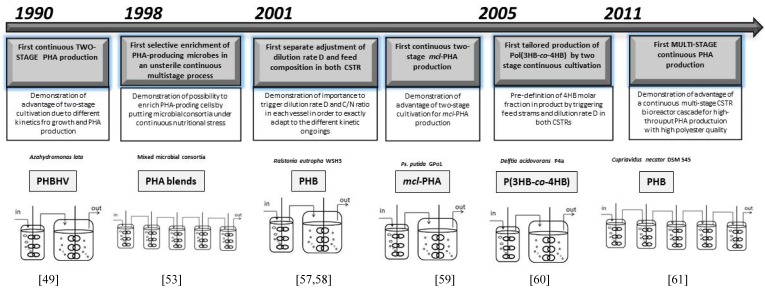
Milestones in continuous process development for PHA production (two- and multistage processes).

Single-, two-, and multistage chemostat set-ups are viable and reported for PHA production. Table 3 summarizes the significant milestones that were reached during continuous single-stage process development for PHA bioproduction, whereas Figure 1 illustrated the history of two- and multistage PHA-production processes. The selection of the adequate number of stages depends on the kinetic characteristic of PHA accumulation by the selected production strain. In the case of non-growth associated or only partly growth-associated PHA production strains, e.g., *Cupriavidus necator*, only minor amounts of PHA are accumulated during non-nutrient limited cultivation. Here, one-stage continuous production does not allow to fully profiting from the biosynthetic potential of the culture, because growth and PHA accumulation are not chronologically separated. This causes incomplete conversion of carbon sources in single stage continuous processes caused by a typically high molar carbon to nitrogen (C/N) ratio in the feed stream; consequently, PHA-poor biomass is produced! If the surplus carbon source in the reactor outlet cannot be recycled to the process, this amounts to a substrate loss and constitutes a considerable cost factor entitled firstly to the direct substrate expenses and, secondly, to the higher costs for treatment of the spent fermentation broth due to an increased (bio)chemical oxygen demand. As a solution, a two-stage process consisting of two continuously stirred tank reactors operated in series (CSTRs) is advised (see later). By using such two-stage systems, microbial growth and product (PHA) formation can be optimized separately and independently from each other. One-stage set-ups are viable and reasonable, e.g., in the case of the strain *Azohydromonas lata*, where significant PHA production already occurs under nutritional balanced conditions in parallel with biomass growth; here, the C/N ratio does not need to be as high as in the case of *C. necator*. But, as shown later, even in the case of *A. lata*, a second stage was demonstrated to be beneficial for optimized PHA productivity; here, the second CSTR provides the cells time of exposure (residence time) needed for complete carbon substrate conversion and high PHA loads. Multistage systems are more complex, more complicated to handle and, due to the high number of connecting devices between the individual CSTRs, feature a higher risk of microbial contamination by infiltration of exogenous cells. Nevertheless, such systems offer the possibility to provide different cultivation conditions in each stage and, with increasing number of stages, approximate the characteristics of continuous plug flow tubular reactors (CPFTRs). As known from the literature, CPFTRs constitute the most suitable process set-up to match the kinetic characteristics of PHA accumulation for most strain/substrate combinations, even better than the second stage of a system of two continuous CSTRs [32]. Further, it is understood that a cascade consisting of five or more CSTRs in series constitute a process-engineering substitute for a CPFTR [32]. Superior product quality can be expected in a CPFTR (or cascade of CSTRs, respectively) by the highly uniform physiological state of the cells; this uniformity is caused by the narrow residence time distribution in such systems [32]. These process-engineering considerations gave the reason for the development of a continuously operated multistage system for PHA biosynthesis at high productivities consisting of five CSTRs in series (5-CSTR) (see later).

**Table 3:** Historical outline of single-stage continuous processes for PHA production.

Year	Strain	Aim	PHA Produced	Significance of the Work for the Scientific Field	Reference
1972	*Azotobacter beijerinkii* NCIB 9067	Investigating the impact of oxygen limitation on PHA synthesis	PHB	First reported continuous PHA production	[53]
1986	*Hyphomicrobium* ZV620	Investigating the impact of carbon to nitrogen ratio and *D* on activity of NH_4_^+^-assimilating enzymes, and on cellular composition (PHB content in biomass)	PHB	Confirmation of significance of carbon to nitrogen ratio to PHA mass fraction in biomass, confirmation of impact of *D* on cellular composition	[54]
1990	*Cupriavidus necator* DSM 545	Increase of PHA productivity and intracellular PHA fraction by continuous operation	PHBHV	First continuous PHA production to enhance product output First continuous copolyester production	[47]
1990	*Haloferax mediterranei* DSM 1411	Monoseptic continuous cultivation of osmophilic strain under unsterile conditions (“septic process”) for high-throughput PHA production	PHBHV	First continuous PHA production under unsterile conditions using extremophiles	[40]
1991	*Ps. putida* GPo1	Continuous production of *mcl*-PHA from fatty acids and octane	*mcl*-PHA	First continuous *mcl*-PHA production	[52,55]
1995	*Cupriavidus necator* DSM 545	Investigating the impact of *D* on polyester composition and productivity	PHBHV	First insights on impact of *D* on PHA production (quality and quantity)	[49]
2000	*Ps. putida* GPo1	Continuous production of *mcl*-PHA from fatty acids under *de facto* C- and N-limitation	*mcl*-PHA	First Dual Nutrient Limited (DNL) continuous PHA production	[50]
2005	*Cupriavidus necator* DSM 545	Continuous production of *scl*-PHA copolyesters with pre-determined monomeric composition	PHBHV	First triggering of *scl*-PHA copolyester composition on the nonomeric level by varying the composition of the continuous feed stream	[43]
2004	*Ps. putida* GPo1	Continuous production of *mcl*-PHA copolyesters with pre-determined monomeric composition	*mcl*-PHA with aromatic building blocks	First triggering of *mcl*-PHA copolyester composition on the nonomeric level by varying the composition of the continuous feed stream First continuous production of an aromatic *mcl*-PHA	[56]
2009	*Ps. putida* GPo1	Production of non-amorphous (crystalline) *mcl-*PHA on a relevant scale	*mcl*-PHA with aromatic building blocks	First continuous production of a crystalline *mcl*-PHA	[45]

## 3. Single-Stage Continuous Systems with Pure Cultures

### 3.1. Scl-PHA Production by Single-Stage Continuous Cultures

Already in the early 1970s, Senior *et al.* acted as pioneers in the field of continuous PHA production. The production strain in the first reported work in this field was a non-capsule forming mutant of the diazotrophic strain *Azotobacter beijerinkii* NCIB 9067. By continuous chemostat cultivations in a CSTR, the impact of limitation of nitrogen, oxygen, and carbon on PHA biosynthesis was investigated. As a major outcome, restricted oxygen supply was the most beneficial factor to boost PHA accumulation, whereas in carbon- or nitrogen-limited cultures, the mass fraction of PHA did not exceed 0.03 g per g CDM in contrast to 0.20 g (at *D* = 0.252 1/h) or even 0.44 g (at *D* = 0.049 1/h) PHA per g CDM in oxygen-limited cultures. These first continuous experiments for PHA production already underlined the importance to adapt τ to the PHA accumulation kinetics in order to obtain satisfying intracellular PHA contents; this is pivotal not only for volumetric PHA productivity, but also to simplify the subsequent PHA recovery from biomass, which is of economic relevance, and for complete substrate conversion. These early continuous experiments should not be interpreted as process development for enhanced PHA production, but were mainly planned to elucidate the biological function of PHA during nutrient limitation. It was revealed that PHA does not only act as carbon and energy storage, but, in *statu nascendi*, also functions as electron sink by converting excess reducing equivalents. Further, this work already describes the risks connected to continuous PHA production by microbial contamination; as a technological solution, the application of thermo-traps to prevent microbial back-growth from the culture to the sterile medium was proposed [53,54].

Due to the restricted interest in the PHA-topic in the 1980s, only one serious report in continuous PHA production was published in literature during this decade; Gräzer-Lampart and colleagues investigated the growth of *Hyphomicrobium* ZV620 and the regulation of different NH4^+^-assimilating enzymes in chemostats supplied with methanol and NH4^+^ as carbon or nitrogen source, respectively, and monitored also PHB accumulation. The authors report a constant carbon content of the cells, independent on *D*, nitrogen, and carbon concentration, and a constant nitrogen and protein content in carbon-limited cultures, whereas in nitrogen-limited cultures, the protein content significantly dropped on expense of increased PHB formation. At D = 0.054 1/h and C/N ratios from 1/12 to 1/22, constant PHB mass fractions of almost 0.3 g/g were reported [54].

About two more decades had to pass before the next continuous process-related studies were published by Ramsay and associates [47]. These authors for the first time proposed continuous cultivation mode as a precious means to maximize PHA productivity and the intracellular PHA fraction. Further, their experiments with different production strains suggest continuous cultivation mode to maximize the 3HV-shares in poly(3-hydroxybutyrate-*co*-3-hydroxyvalerate) (PHBHV) copolyesters in order to trigger the material properties. This is a decisive aspect considering the fact that, in discontinuous cultivations, 3HV-related precursor substrates like propanoate or pentanoate inhibit growth of the production strain already at rather low concentration levels. Economically, conversion yields of these expensive precursors to 3HV are decisive for the cost of the final bioproduct. By one-stage chemostat (*D* = 0.15 1/h) cultivation on glucose, *C. necator* DSM 545 produced 5 g/L of biomass with a PHB mass fraction of 0.33 g/g. *A. lata* ATCC 29714 cultivated on sucrose at the same *D* gave similar results; here, CDM and PHB mass fraction amounted to 4 g/L and 0.40 g/g, respectively. When cultivated on glucose and staged propanoate concentrations in the feed stream (up to 5 g/L), *A. lata* accumulated PHBHV with 3HV fractions of up to 0.20 mol 3HV per mol PHA. Replacing 3 g/L propanoate by 3 g/L pentanoate caused production of the same total PHBHV concentration, but higher 3HV shares in PHBHV (0.15 mol per mol *vs.* 0.38 mol per mol, respectively) [47].

A number of additional studies are available for PHBHV production by *C. necator* in one-stage continuous cultures. In 1995, Koyama and Doi reported the synthesis of PHBHV in a 2.5 L bioreactor using fructose (17.5 g/L) and pentanoate (2.5 g/L) in the carbon feed. A maximum PHA productivity of 0.31 g/(Lh) was attained by running the process with a *D* of 0.17 1/h. Stepwise increasing *D* from 0.06 1/h to 0.32 1/h changed the 3HV fraction in PHBHV from 0.11 to 0.79 mol per mol. At the same time, CDM and total PHA concentration decreased significantly. This underlined firstly that the cells require longer exposure time (higher τ) for both growth and PHA accumulation, and, secondly, confirms the faster consumption of pentanoate than fructose. As the first report for dependence of PHA properties on the applied *D* regime, the work shows that molar mass of the polyester can be increased in parallel with higher *D*, but at the expense of the overall PHA productivity [49].

About ten years later, Yu and colleagues used different mixtures of glucose and propanoate at staged *D* to scrutinize one-stage continuous PHBHV production by *C. necator*. The authors revealed the direct correlation of the molar 3HV fraction in PHBHV and the proportion of propanoate and glucose in the substrate feed. PHBHV with 3HV fractions between 0.40 and 0.60 mol/mol were obtained using mixed feeds of glucose (10 g/L) and staged propanoate concentrations (7 to 15 g/L). Propanoate concentrations below 5 g/L resulted in setting of stable steady states at *D* up to 0.15 1/h, whereas maximum PHA productivities where reported at *D* = 0.10 1/h. Propanoate at concentrations exceeding 7 g/L prevented the setting of steady states; at a rather low *D* of 0.028 1/h, it was possible to reach a steady state providing propanoate concentrations not exceeding 7 g/L [48].

### 3.2. Mcl-PHA Production by Single-Stage Continuous Cultures

In 1991, Ramsay and colleagues described the first continuous medium chain length PHA (*mcl*-PHA) biosynthesis by *Pseudomonas putida* GPo1 (original name in article “*Ps. oleovorans* ATCC 29347”). This study was motivated by the strong growth inhibition of many structurally related substrates needed for *mcl*-PHA biosynthesis, mainly fatty acids like octanoate in the case of the reported experiments. The authors suggested continuous cultivation as a way to provide the cells with sufficient substrate without exceeding inhibiting substrate concentrations in the culture broth. At a *D* of 0.24 1/h, *mcl*-PHA copolyesters with constant shares of four different monomeric building blocks, namely 3HB, 3-hydroxyhexanoate (3HHx), 3-hydroxyoctanoate (3HO), and 3-hydroxydecanoate (3HD), was produced under nitrogen limited conditions; the intracellular mass fraction amounted to 0.13 g *mcl*-PHA per g CDM. Additionally, also octane was investigated as carbon source, but rather modest results were obtained due to the high hydrophobicity of this substrate, which counteracts its susceptibility by the cells [55]. It appears likely that the droplet size in this study was not small enough, since a direct relationship between growth and stirrer speed/surfactant could be identified by Schmid and colleagues [62].

Later, the same research group applied hexanoate and octanoate for continuous *mcl*-PHA production by using *Pseudomonas resinovorans* as production strain. Using a fixed *D* of 0.25 1/h (octanoate as substrate) and 0.125 1/h (hexanoate as substrate), different C/N ratios in the feed stream were tested. Under nitrogen-limited conditions, the molar fractions of building blocks in the produced *mcl*-PHA (3HB, 3HHx, 3HO, and 3HD) remained constant [63]. In accordance to the previously summarized outcomes using *Ps. putida* GPo1 [55], the chain length of the predominant monomeric building block equaled the chain length of the substrate (3HHx in the case of hexanoate, 3HO in the case of octanoate). For the hexanoate case, the authors did not report data for productivity, nor for PHA concentration, nor for PHA mass fractions in CDM in cells or PHA concentration due to the modest quantities of product. Different to the results obtained with *Ps. putida* GPo1 on octanoate [55], *mcl*-PHA productivity was enormously boosted in *Ps. resinovorans* by nitrogen limitation. Yet specific *mcl*-PHA productivity on octanoate under comparable conditions of *D* and substrate concentration was still substantially lower for *Ps. resinovorans* (0.50 g/(gh)) than for *Ps. putida* GPo1 (0.74 g/(gh)) [63].

A Swiss group investigated one-stage continuous cultivation for production of *mcl*-PHA by *Ps. putida* GPo1 after a thorough optimization of the medium composition concerning the concentration of nitrogen, phosphate, iron and magnesium [52]. Based on the capability of this strain to metabolize alkanes towards *mcl*-PHA (see also [64]), gaseous octane was provided as carbon source and supplied to the cells by saturating the aeration stream with this alkane. Under nitrogen limited conditions and a *D* of 0.2 1/h, *mcl*-PHA consisting of 3HHx and 3HO were produced at maximum productivities of 0.17 g/(Lh). By boosting the cell density by increased nitrogen supply at the same *D*, productivity was improved to 0.58 g/(Lh). Although the intracellular PHA fraction remained rather low (only 0.23 g PHA per g CDM), the continuously operated process remained stable for about one month. By further process development, especially in terms of media composition and bioreactor mixing, volumetric productivity was even increased to 0.76 g/(Lh) [52].

By further investigating different C/N ratios in single-stage chemostat cultures of *Ps. putida* GPo1 at fixed *D* of 0.2 1/h, Durner and associates described for the first time so-called “dual nutrient limited growth conditions” (DNL), which can be understood as an intermediate growth regime of coinciding nitrogen and carbon limitation, and the benefit of its use for enhanced *mcl*-PHA production [50]. In contrast to nitrogen-limited cultures, neither nitrogen nor carbon source is actually detectable in the fermentation broth of DNL cultures according to the authors. It has to be noticed that the substrates can become detectable under carbon limitation according to Monod. Consequently, this statement is only valid for lower dilution rates. The authors reasoned that DNL conditions constitute the optimum strategy to produce PHA from growth-inhibiting substrates by providing the cells with the adequate amount of the substrate without impairing the cell activity. Hexanoate, heptanoate, octanoate, and nonanoate were applied as carbon sources. Again, copolyesters were obtained in which the chain length of the predominant monomeric building block equaled the chain length of the substrate, the rest to its products of the ß-oxidation of the alkanoate. Using octanoate, cells harboring 0.42 g *mcl*-PHA per g CDM were obtained under nitrogen-limited conditions; this is superior to the values obtained for heptanoate and nonanoate (around 0.30 g/g), and especially to the use of hexanoate (0.006 g/g). Under carbon limited conditions, only about 0.02 g *mcl*-PHA per g CDM were detected using heptanoate and octanoate, and around 0.19 g/g using nonanoate; using hexanoate, no detectable quantities of PHA were produced at all. For octanoate, it was clearly demonstrated that, if tailoring DNL conditions, the C/N ratio has to approach the frontiers to nitrogen-limiting conditions, but not exceeding a value resulting in detectable substrate concentrations in the fermentation broth. Because all carbon sources are completely utilized during DNL cultivation, the authors realized the potential of DNL cultivation for production of structurally tailored PHA copolyesters based on multiple carbon sources. By triggering the ratio between carbon sources according to the desired polyester composition, copolyesters of constant monomeric composition are generated during steady state [33,50].

Based on these findings, *C. necator* DSM 428 was cultivated for short chain length PHA (*scl*-PHA) production in a chemostat under DNL conditions with mixtures of butanoate and pentanoate as carbon source. By simply changing the ratio butanoate/pentanoate and, at the same time, keeping the total C/N ratio constant at 17/1, conditions beneficial for high PHA synthesis were provided. All experiments were operated at a *D* of 0.1 1/h. Butanoate as the sole carbon source resulted in PHB homopolyester synthesis, whereas increasing pentanoate shares resulted in synthesis of PHBHV copolyesters with increasing 3HV fractions. Using pentanoate as sole carbon source, the molar 3HV fraction in PHBHV amounted to 0.62 mol/mol. The products were isolated and revealed high molar mass at low PDI; melting points (*T_m_* between 178 °C for PHB and 80 °C for PHBHV with 0.62 mol 3HV per mol PHA) and glass transition temperatures (*T_g_* between 5.9 °C for PHB and −4.1 °C for PHBHV with 0.62 mol 3HV per mol PHA) decreased tremendously with increasing 3HV fractions. These findings confirmed that DNL continuous process was also suggested as a tool to trigger the composition of PHBHV copolyesters exacter than possible in only nitrogen limited continuous cultures [43]. Similar to the outcomes with *Ps. putida* GPo1 [50], DNL growth is useful because the cell’s metabolism is not derogated when the substrate is completely converted, or its concentration is kept below an inhibiting concentration level [38,51]. Further investigations should investigate the impact of different *D* on the monomeric polyester composition, especially for high pentanoate shares in the feed stream.

The viability of tailored PHA synthesis by applying multiple carbon sources under DNL in one-stage chemostats was also demonstrated for *mcl*-PHA production by *Ps. putida* GPo1. Here, a *mcl*-PHA harboring monomers with unsaturated side chains was produced by co-feeding of octanoate and 10-undecenoate. *D* amounted to 0.1 1/h, and the applied C/N ratio of 16/1 resulted in DNL under steady state conditions. A share of 10% 10-undecenoate in the feed mix resulted in production of *mcl*-PHA with 10% unsaturated monomers, whereas 25% of unsaturated building blocks were detected using 25% of 10-undecenoate in the feed. Due to the catabolism of the substrates to one or more acetyl-CoA units during ß-oxidation, the resulting polymers consisted of 3HHx, 3HO, 3-hydroxy-undec-10-enoate, 3-hydroxynon-9-enoate, and 3-hydroxyhept-7-enoate units. For the polymer containing 10% unsaturated building blocks, molar mass was reported with 113,000 at a PDI of 2.02. After extraction, this *mcl*-PHA signified its high suitability for post-synthetic chemical modification; coatings against bio-fouling where produced by linking this “functional” *mcl*-PHA to the bioactive compound zosteric acid [44]. It should also be mentioned that high cell density chemostats require high oxygen transfer rates. This problem was addressed in a high pressure bioreactor, where cells were literally “put under pressure”. No influence of enhanced oxygen tension on the content of unsaturated PHA was observed. However, the oxygen transfer would be sufficient to provide an elevated cell density yielding in a volumetric PHA productivity of about 11 g/(Lh) [65].

Even structurally exotic *mcl*-PHAs were produced by applying multiple carbon substrates in DNL continuous cultivation of *Ps. putida* GPo1. At a *D* of 0.1 1/h and molar C/N ratios in the feed stream of 15/1, different mixtures of 5-phenylpentanoate, octanoate, and 10-undecenoate were used for production of diverse poly(3-hydroxy-5-phenylvalerate-*co*-3-hydroxyalkanoate-*co*-3-hydroxy-ω-alkenoate)s; the molar fraction of aromatic monomers ranged from 0 to 0.52 mol/mol. With increasing aromatic (3-hydroxy-5-phenylpentanoate) content, *T_g_* linearly increased from −37.6 °C to −6 °C [56]. Further, DNL growth was also applied to co-supplement a continuous chemostat cultivation based on lactic acid with the aromatic co-substrate *p*-methylphenylvaleric acid, resulting in a crystalline poly(3-hydroxy-*co*-*p*-methylphenylvalerate) *mcl-*PHA which is the first report on continuous production of a non-amorphous *mcl*-PHA [45].

In a recent study carried out by Kedia and colleagues, volatile fatty acids (VFAs) from anaerobic fermentations have been applied as carbon substrate for single-stage, *de facto* continuous, PHA production by *C. necator*. It turned out that a number of factors, mainly the feeding regime, are decisive for the conversion efficiencies of VFAs to PHA. When VFA were supplied as single continuous feed, concentrations higher than 20 g/L VFA resulted in substrate inhibition; in this case, low conversion rates of VFA into PHA were observed. Merely 18% of acetate and 12% of butyrate was metabolized to PHA, resulting in rather low mass fractions of less than 0.65 g PHA per g CDM. An optimized automatic VFA feeding regime was coupled to the pH-value control of the medium, hence, depletion of carbon sources resulted in an increase of the pH-value, which provoked an addition of acids, thus leading to a *de facto* continuous feeding regime. A feedback cultivation system like this is usually also termed as “pH-auxostat”. The conversion of VFA to PHA was almost doubled to 0.33 g/g for acetic acid and 0.22 g/g for butyrate, respectively; extraordinarily high PHA loads up to 0.75 g PHA per g CDM were obtained under such quasi-continuous conditions [66].

## 4. Two-Stage Continuous Systems with Pure Cultures

### 4.1. Scl-PHA Production by Two-Stage Continuous Cultures

As detailed in the prior paragraphs, all single-stage experiments with the most powerful strains *C. necator* or *Ps. putida* resulted, in addition to incomplete carbon source conversion, in rather low biomass concentration and low intracellular PHA fractions; DNL cultivation constitutes an exception for the application of inhibiting C-sources, but not the method of choice for bulk-production of simple *scl*-PHA from non-toxic substrates. In order to achieve high-throughput production of catalytically active biomass, as a prerequisite for high productivity of the entire process, high *D*, approximating the µ*_max._* value, the so-called *D_opt._*, is needed. At the same time, running the process at high *D* results in decreased intracellular PHA fractions due to the lacking τ needed by the strains for complete carbon utilization. These are expected findings, since these strains accumulate PHA predominately in a non-growth-associated mode. This means that the phase of formation of active biomass under balanced nutritional conditions can be clearly distinguished from the subsequent phase of predominant diverting of the intracellular carbon flux towards PHA formation; hence, kinetics for growth and PHA-accumulation are different! Here, single-stage continuous set-ups always require a compromise between productivity, concentration and intracellular PHA fraction [39]. For such cases, optimal conditions for both cell growth and PHA production cannot be maintained in single-stage continuous systems; a multistage system should be more suitable for this purpose. Therefore, Ramsay and colleagues demonstrated for the first time the advantages of two-stage chemostat PHA production to one-stage set-ups. These authors investigated the first two-stage chemostat process for PHBHV production using *A. lata* on sucrose and propanoate. In the first stage (*D* = 0.15 1/h), propanoate was completely consumed, but detectable concentrations of sucrose remained. In this first stage, 0.48 g PHA per g CDM with 0.185 mol 3HV per mol PHA were continuously produced; subsequently, the fermentation broth was transferred into the second bioreactor (*D* = 0.15 1/h). Here, remaining sucrose from the first stage was completely consumed, and 0.58 g PHA per g CDM with 0.11 mol 3HV per mol PHA was obtained. The experiment also showed the inhibition of sucrose uptake by propanoate concentrations in the feed exceeding 8.5 g/L [47].

*Ralstonia eutropha* WSH3 was cultivated by Du and associates in two CSTRs in series. The reactor volumes amounted to 1.2 L in the first stage and 1.4 L in the second stage. Using nitrogen-rich and carbon-limited conditions, mainly active PHA-poor cells were produced in the first stage; here a maximal CDM of 27.1 g/L was obtained at a *D* of 0.21 1/h. By applying nitrogen-limitation and sufficient glucose in the second stage, PHB production was favored, and a maximal PHA concentration of 47.6 g/L at a *D* of 0.14 1/h was obtained. The maximal PHA productivity reached 1.43 g/(Lh) at a *D* of 0.12 1/h with a rather modest intracellular PHB fraction of around 0.48 g per g CDM. Maximal yields for PHA synthesis from glucose (0.36 g/g), together with excellent PHA productivity (1.23 g/(Lh)) and 0.72 g PHA content per g CDM were obtained at a *D* of 0.075 1/h [57]. The set-up resulted in respectable results for CDM, PHB fraction and productivity, but the optimum values for these parameters were not obtained simultaneously, hence not with the same *D*. This means that the authors did not fully exploit the potential of the equipment. After the experiments, kinetic analysis of the two-stage continuous process was carried out to explicitly study the kinetic particularities of both microbial growth and PHA formation under steady state conditions. This analysis exposed that microbial growth in the first stage can accurately be described by the kinetic model according to Monod, with a washout to be expected by excessive *D* above 0.40 1/h. Specific PHA synthesis rate in the second vessel was shown to be highly dependent of the C/N ratio, and was also in good agreement with the Monod model. The maximum specific PHA production rate (*q_Pmax._*) for nitrogen-limited conditions amounted to 0.18 g/(gh) [58].

Only few additional studies in a two-stage continuous *scl*-PHA production are available using new microorganisms. In two CSTRs in series, Mothes and Ackermann farmed *Delftia acidovorans* P4a on mixtures of acetate and the 4-hydroxybutyrate (4HB)-related co-substrate γ-butyrolactone (GBL). Dependent on the ratio of acetate and GBL in the feed stream, cells accumulated poly(3HB-*co*-4HB) copolyesters with a 4HB fraction of 0.027 to 0.19 mol 4HB per mol PHA. *D* was adjusted to 0.2 1/h for the first, and, in order to provide higher τ, to 0.06 1/h for the second CSTR. It was demonstrated that the optimum PHA productivity and the pre-definition of 4HB contents in PHA can be achieved by triggering the ratio between acetate and GBL [60]. Similar to the findings described before for the substrate system butanoate and pentanoate [43], also this example of application of inhibiting substrates (acetate and GBL) demonstrates the viability of continuous production mode especially to conveniently supply the cells with the required amounts of substrates without exceeding inhibiting concentration levels.

### 4.2. Mcl-PHA Production by Two-Stage Continuous Cultures

As continuation of the previous single-stage continuous investigations [63,64], *Ps. putida* ATCC 29347 was used for biosynthesis of *mcl*-PHAs from gaseous octane in a continuous system of two serial CSTRs of 3 L volume each. Also here, the separation of the process in a first stage (biomass production at high specific growth rates of *D* = µ*_max._* = 0.21 1/h) and a second stage (PHA production at extended exposure time at *D* = 0.16 1/h) successfully addressed the kinetic particularities. After optimizing *D*, temperature, pH-value, growth-limiting component, and carbon source, a volumetric PHA productivity of 1.06 g/(Lh) and a fraction of 0.63 g PHA per g CDM were obtained in the second CSTR. The authors underlined, due to the completely different kinetics of biomass growth and *mcl*-PHA accumulation, the superiority of two-stage processes to single-stage set-ups, particularly for *mcl*-PHA production [59]. This was again confirmed later by using octanoate as sole carbon source for *Ps. oleovorans*; here, *mcl*-PHA accumulation is maximal (mass fraction of 0.63 g per g CDM, volumetric productivity 1.06 g/(Lh)) when the cells are cultivated at a *D* of 0.22 1/h in the first stage, and 0.16 1/h in the second stage, whereas µ*_max._* of this strain amounts to 0.48 1/h as reveled by single-stage chemostat cultures. [46]. It should be noted here that it was attempted to produce block co-polymers (*b-*PHA) by the two-stage chemostat of *Ps. putida* GPo1. Nevertheless, the strain accumulated two different polymers under the given conditions. Thus, whether really blocky-structured PHA can be produced in multi-stage chemostat is questionable, and should be the subject of experimental investigations.

## 5. Multistage Process with Pure Cultures

A novel strategy to optimize PHA production both in terms of quality and quantity was elaborated by Atlić and associates [61]. These authors applied a 5-CSTR bioreactor cascade; as mentioned before, this cascade can be considered a flexible process engineering alternative to a CPFR [32]. The first stage of the 5-CSTR (7.5 L bioreactor, 1 L working volume) operated under nutritionally balanced conditions and served for balanced microbial growth to produce high concentration (about 20 g/L) of active, PHA-poor biomass. Next, the fermentation broth was continuously transferred from the first into the subsequent CSTRs (3.6 L bioreactors each, 1 L working volume), where PHA accumulation was boosted by nitrogen limitation under continuous supply of carbon source (glucose) to each vessel. As production strain, *C. necator* DSM 545 was used in these experiments [61]. The developed PHA production process was characterized by high productivity and high intracellular PHA fraction. The results obtained by the 5-CSTR confirmed theoretical considerations concerning its potential regarding high volumetric and specific productivity (1.85 g/(Lh) and 0.100 g/(gh), respectively), high intracellular PHA fraction (0.77 g/g) and PHA concentration (63 g/L). In addition, the isolated PHA samples reveled high quality (molar mass of 665,000, PDI = 2.6). The 5-CSTR was operated under stable steady-state conditions for extended time periods exceeding 200 h. By carefully adapting the glucose supply to its conversion rate, only negligible glucose concentrations remained in the outlet stream from the fifth CSTR, resulting in a low pollution of the spent fermentation broth to be disposed of later. The authors emphasized that implementing a 5-CSTR for PHA production might result in an economic process and suggested that, based on the system’s flexibility, the product properties are tunable by regulating the polymer composition by different co-substrate additions to each CSTR [61]. This should even enable the production of *b-*PHA with high microstructure diversity. These materials comprise alternating soft and hard segments, e.g., 3HB and 3HV blocks, 3HB and 4HB blocks, 3HB and 3HHx blocks, 3-hydroxyproionate (3HP) and 4HB blocks, or 3HHx and 3HD-*co*-3-hydroxydodecanoate blocks with superior properties, especially regarding tensile strength and elongation to break. It was shown that such *b-*PHAs are produced by mutants of *Ps. putida* [67,68,69,70,71]. Until now, *b-*PHAs were not manufactured on a relevant scale; by using a multi-stage continuous approach, they should finally be accessible at sufficient quantity, although the feasibility of this concept still needs experimental proof.

Mathematical modeling becomes an increasingly precious tool firstly to describe PHA microbial PHA productions processes, and, secondly, to restrict the number of experiments during process development. As an example, the difficult kinetic processes in the described 5-CSTR gave Horvat and colleagues reasons to establish a formal kinetic mathematic model of this process. The aim of this model was the spotting of possibilities to even better profit from the 5-CSTR system by further increasing PHA productivity and intracellular PHA content. Partially growth-associated PHA production under nitrogen-limited growth, typical for *C. necator*, was chosen as modeling foundation, based on the Luedeking-Piret’s model of partial growth associated product synthesis. Specific growth rate relations adjusted for dual substrate (C and N source) limited growth according to Megee *et al.*, and the Mankad-Bungay relation were tested. The first stage of the 5-SCR was modeled according to a nutritionally balanced continuous biomass production system, the second as dual-substrate controlled process, while the three subsequent reactors were supposed to produce PHB under carbon-rich and nitrogen-limited conditions. Simulated results for production optimization obtained by the applied models and *in silico* optimization suggest that PHB productivity of the whole system could be enormously boosted to 9.95 g/(Lh) by optimizing experimental conditions regarding the overall *D*, C and N source feed concentration [28]. Based on this low-structured model by Horvat *et al.*, Lopar and colleagues established a high structured metabolic model for 5-SCR PHB synthesis by *C. necator* on glucose [72]. This complex model consisted of 43 mass balance equations related to 43 intracellular metabolites, and comprises one of the most detailed kinetic insights into PHA biosynthesis. The metabolic states of cells cultivated in the 5-CSTR were analyzed using elementary flux modes and two-dimensional yield space analysis. Two different carbon source feeding regimes were investigated. Regarding PHB and biomass yields, values of the more advantageous feeding strategy were used as the data source for elementary modes and metabolic flux calculations. Metabolic fluxes were calculated from experimental yield data by using a combination of elementary modes. The high structured metabolic model was validated by comparison of experimental data from 24 h batch cultivation and simulated results for confirmation of its robustness [72].

## 6. Strategies for Continuous Enrichment of PHA-Accumulating Organisms

It was demonstrated by several groups of authors that PHAs can be produced by open mixed cultures providing an appropriate enrichment step based on PHAs biological function. Hence, nutritional stress conditions are provided by depletion of exogenous carbon source; this provides PHA-harboring cells an advantage for survival.

This was proven in a sequencing batch reactor used to enrich a mixed culture of cells able to produce PHA. The set-up was operated with 24 h residence time for biomass and feast−famine cycles of 12 h; acetate acted as carbon feed. In subsequent growth-limited fed-batch experiments, the enriched mixed culture accumulated PHA up to a mass fraction of 0.89 g PHA per g cell dry mass (CDM) after 7.6 h of cultivation at an average specific productivity of 1.2 g/(gh). The produced PHA was identified as the homopolyester poly(3-hydroxybutyrate) (PHB). The predominant species of the culture was an unknown *Gammaproteobacterium* with little similarity (based on 16S rRNA analysis) with known bacterial species. This mixed culture process for PHA production was run without any sterility precautions, hence, without aseptic conditions. On the raw material side, the authors suggest the application of waste streams rather than pure carbon substrates [73].

Similar investigations for PHA production with mixed microbial cultures were recently performed by Villano and associates. These authors used a sequential process consisting of three different stages, namely a sequencing batch reactor for mixed culture selection, a reactor for PHA accumulation and, finally, a facility for polymer extraction. The entire set-up was operated continuously for at least four months to test the process robustness and the related variability of the accumulated PHA in terms of monomeric composition, molar mass distribution, and thermal polymer properties. By operating the first and second stage with high concentrations of a mixture of acetate and propanoate (8.5 g and 29.1 g COD/(Ld)), 1.43 g/(Ld) PHA were produced continuously and at stable rates. The third vessel was operated with two different agents (NaOH or NaClO) for biomass digestion for PHA recovery. Despite the stable operating conditions and performance, a considerable fluctuation of the 3-hydroxyvalerate (3HV) content in the polyester between 0.04 and 0.20 g/g (after second reactor) and between 0.09 and 0.13 g/g (after extraction vessel), respectively, was observed. The molar mass of the produced PHA ranged between 340,000 and 540,000 with a rather high polydispersity index (PDI). In contrast, thermal polymer properties (melting temperature, glass transition temperature, crystallinity, *etc.*) did not significantly fluctuate over time, but, as expected, were highly influenced by the selected extraction agent (NaOH or NaClO) [42].

Similarly, Moralejo Garate *et al.* explored the potential of a mixed microbial community for PHA production from glycerol. Here, a PHA-producing microbial community was enriched based on a feast-famine cultivation regime. A sequencing batch reactor supplied with a glycerol feed was operated at a residence time τ of two days and with feast-famine cycles of 24 h. This set-up was used to enrich a mixed community of PHA-producing microbes. In a subsequent fed-batch PHA production step under growth-limiting conditions, the enriched mixed community produced PHA up to a mass fraction of 0.8 g PHA per g CDM. The results indicated that the feast-famine-based enrichment strategy might be a feasible tool to enrich bacteria able to metabolize glycerol to PHA [74]. These investigations were later intensified to understand the effect of the cycle length on the bacterial enrichment process with emphasis on the distribution of the glycerol flux towards PHA or competing intracellular products. For this purpose, two sequencing batch reactors where operated with the same τ. As major result, a long cycle length of 24 h was beneficial for optimum PHA accumulation, whereas significantly shorter cycle lengths favored the formation of competing storage products like poly(glucose) [75].

An unsterile multi-stage approach for selective enrichment of PHA accumulating strains from environmental samples by long-term carbon starvation was already described by Renner and colleagues. In principle, this approach profited from the survival advantage of PHA-harboring cells under exogenous carbon-deficient conditions as selection criterion. The authors supplied the first vessel of a 5-CSTR with a carbon-rich and nitrogen-poor medium, and operated the system at extremely low dilution rates (*D*). Starting from the second vessel, the cells were exposed to both carbon- and nitrogen-limited conditions, resulting in a dying off of those cells which did not accumulate PHA in the first vessel. The total operation time under both nitrogen and carbon limited conditions lasted up to 200 h. Starting from various investigated environmental samples, a total of nine microbial strains of differently pronounced capability to produce PHA were enriched [76].

## 7. Unsterile and Open Continuous Processes for PHA Production

In the field of extremophile PHA production in discontinuous mode, especially the application of extremely halophile archaea and eubacteria is intensively studied [77,78,79,80]. The application of such extremophile organisms for continuous cultivation can be regarded as a feasible tool due to the low risk for microbial contamination. As described in the next paragraphs, even open and/or unsterile continuous processes are described using such strains; this can be regarded as an important step towards energy-efficient PHA synthesis.

Already in 1990, Lillo and Rodriguez-Valera studied PHA production by the highly halophilic archaeon *Haloferax mediterranei*, a member of the extremely salt-requiring branch of halobacteria. This strain needs a minimum of 1.5 M NaCl for growth and tolerates up to 5 M NaCl in the cultivation medium, which corresponds to an impressive salt lot of about 300 g/L [40]. Among all archaea, this strain displays the highest reported intracellular PHA fractions; in optimized fed-batch cultures, intracellular PHA mass fraction of more than 0.8 g per g CDM were reported [81]. Continuous one-stage cultivation of *H. mediterranei* as demonstrated by the authors was possible due to the fact that the strain accumulates considerable amounts of PHA in a growth-associated way already under nutritionally balanced conditions. As a particularity, the strain produces 3HV-containing copolyesters from structurally unrelated carbon sources like glucose, fructose, sucrose, starch, or inexpensive waste materials like whey, crude glycerol, rice-based ethanol stillage, vinasse, *etc*. [81,82,83,84,85]. The enzymatic backgrounds of this fascinating ancient PHA-producer, and ways to boost its PHA production potential were elucidated only during the last few years [86,87,88,89,90]. Depended on the feeding strategy, blocky structured (*b-*PHA) or random PHBV copolyesters are produced by this strain [87]. Additional benefits of *H. mediterranei* for industrial PHA manufacture, namely a convenient solvent-free and low-energy downstream processing, as well as the drawbacks arising from the high salinity of the nutritional medium that demands high requirements to the bioreactor equipment and disposal of spent fermentation broth were comprehensively summarized before [91]. Only recently, strategies to recycle the highly saline side streams of *H. mediterranei* fermentations were reported [92]. Grace to controlled batch-experiments done by Lillo and Rodriguez-Valera on bioreactor scale it is known that, in contrast to phosphate limitation, restricted supply of nitrogen or oxygen are not feasible to boost PHA biosynthesis in *H. mediterranei* [40]. In continuous bioreactor experiments, the authors provided glucose as carbon source together with two different phosphate concentration levels. For each phosphate concentration, five different *D* levels between 0.02 and 0.10 1/h were studied. Best results were reached at *D* = 0.02 1/h with a mass fraction of 0.42 g PHA per g CDM, a PHA concentration of 1.5 g/L, a volumetric productivity of 0.03 g/(Lh), and a specific productivity of 0.014 g/(gh). To test the robustness and long-term stability of the organism, the process was repeated by taking only minimal sterility precautions. Operated at a high *D* of 0.12 1/h, the continuous process maintained contamination-free for three months [40].

A halophile and at the same time alkalophile bacterium termed *Halomonas campaniensis* LS21 was isolated and investigated as potential production strain for a seawater-based open, unsterile and continuous PHA production process based on mixed substrates mainly containing polysaccharides, lipids and proteins. To investigate the viability of open and long-term cultivation as well as genetic manipulation of this organism, PHB was selected as model product for the application of *H. campaniensis* LS21 in a long-term continuous cultivation process. Wild type and recombinant *H. campaniensis* LS21 harboring the PHB synthesis genes (*phbCAB*) were continuously cultivated for 65 days in artificial seawater containing mixed substrates mimicking kitchen waste. Under halophile (27 g/L NaCl) and alkaline (pH-value 10) conditions at 37 °C, the recombinant strain accumulated about 0.7 g PHB per g CDM, in contrast to 0.26 g per g produced by the wild type strain; both cultures remained monoseptic. The strain excretes extracellular hydrolytic enzymes during the whole process in parallel to growth, thus enabling the consumption of the mixed substrates. The recombinant strain stably maintained the plasmid encoding for PHA-synthesis enzymes over the entire 65 days process. The authors suggest that, combined with its convenient susceptibility for genetic manipulation, *H. campaniensis* LS21 could provide a powerful platform for low-cost and low-energy production of PHA and other chemicals from inexpensive feedstock [93].

The next unsterile and continuous fermentation process was developed based on another halophilic bacterium, namely *Halomonas* TD01 originally isolated from a Chinese salt lake. It can be cultivated to cell densities up to 80 g/L CDM containing 0.80 g PHB per g CDM in glucose-based 56 h fed-batch fermentation set-ups. In a 14-day open, unsterile, and continuous two-stage process, CDM reached an average of 40 g/L containing 0.60 g PHB per g CDM in the first reactor containing a saline glucose and nitrogen medium. Continuously transferring the fermentation broth from the first to the second reactor containing a nitrogen-free saline glucose medium had two major effects: On the one hand, this transferring diluted the cell concentration, but, on the other hand, resulted in a constant PHB mass fraction between 0.65 and 0.70 g PHB per g CDM. Glucose conversion yields amounted to 0.20 to 0.30 g PHB per g glucose in the first reactor, and astonishingly exceeded even 0.50 g per g in the second one. This unsterile, open, and continuous fermentation process might provide the next decisive step towards cost-efficient PHA production [94]. Later, *Halomonas* TD01 was genetically engineered by a gene knockout procedure. By deleting the gene encoding 2-methylcitrate synthase, the conversion of propanoate to 3HV in random PHBHV copolymers was increased to almost 100%. In a glucose mineral medium containing 0.5 g/L propanoate, cells accumulated 0.70 g PHBV per g CDM with 0.12 mol 3HV per mol PHBHV. Deletion of PHA depolymerase genes was also accomplished in order to prevent intracellular PHA degradation during industrial fermentation process; unfortunately, the PHA content could not be significantly enhanced. In 500 L pilot-scale studies, the engineered *Halomonas* TD01 reached 112 g/L CDM containing 0.70 g PHB per g CDM, and to 80 g/L CDM with 0.70 g PHBHV (molar 3HV fraction 0.08 mol/mol) in the presence of propanoate. In shaking flask experiments, even 0.92 g PHB per g CDM was reached with significantly enhanced conversion yield for glucose to PHA [95]. Further experiments to increase the genetic engineering stability of *Halomonas* TD01 involved the partial inhibition of the DNA restriction/methylation system. In addition, a stable and conjugative plasmid pSEVA341 with a high-copy number was constructed to induce the expression of multiple pathway genes. Combined with the insertion of 2-methylcitrate synthase and depolymerase deletion, the new construct termed *Halomonas* TD08 was able to accumulate up to 0.82 g PHBHV per g CDM [96]. Stability and performance of all these new genetically engineered strains with highly promising features according to discontinuous investigations have now to be tested under continuous operational conditions.

## 8. Conclusions

Based on the selected case studies with different microbial production strains, the review article reveals the undisputed potential of chemostat continuous cultivation to make processes for PHA biosynthesis more efficient in terms of productivity and product quality, and increased efficiency of the downstream processing. It was shown that bringing in line the process design and kinetic particularities of selected strain/substrate pairings are pivotal to developing an economically efficient process characterized by, e.g., excellent volumetric productivity and a substrate conversion as complete as possible.

High stability of the microbial production strain is definitely the condition *sine qua non* to ensure long-term stability of the continuous process. Enrichment and exploration of novel powerful PHA producers especially suitable for long-term cultivation appears as the method of choice for a quick industrial breakthrough of continuous PHA production. Fortunately, such strains are also found among diverse extremophile genera, as successfully shown by stable cultivations under unsterile conditions with halophile archaea and eubacteria. These strains should exhibit unchanged metabolic characteristics during extended processes time, and, under given operational conditions, faster growth if compared to precarious microbial contaminants. In terms of sustainability, technical feasibility and public and legal acceptance, this approach should be superior to alternative genetic engineering approaches to increase the stability of established production strains or to continuous antibiotic dosage to protect cultivation set-ups. In addition, the medium composition plays an important role in continuous cultivations.

As the next step, inexpensive carbon substrates should be tested under continuous conditions in order to further minimize PHA production costs. Such feedstock is found among waste and surplus streams from diverse industrial branches; the viability of their application in continuous mode was already reviewed in this article for crude glycerol from biodiesel production. Many additional inexpensive substrates, such as whey from dairies, hydrolysates of lignocellulosics, waste lipids, molasses, *etc.*, are anticipating their testing as PHA-feedstock in continuous mode. As an unprecedented approach, continuous PHA-production by cyanobacteria should be investigated both under photoautotrophic and mixotrophic conditions; this approach would benefit from a sequestration of industrial effluent CO_2_ and chemoorganic industrial waste streams, but still needs technological development on the process engineering side, deeper understanding of phototrophic PHA biosynthesis, and the due identification of the most promising cyanobacterial PHA producers.

R&D efforts should also be devoted to fully benefit from the possibilities of continuous PHA production for designing of novel biopolymers of custom-made composition on the molecular level in order to fine-tune material characteristics. The production of blocky-structured co- and terpolyesters in a multistage CSTR cascade should provide the possibility to manufacture new biopolymers displaying enhanced properties. This would contribute to a quick market penetration of biopolymers by precisely meeting the customer’s demands of product performance and, to a relevant extent, eliminate petrol-based plastics from the market.
